# Advancing the science of qualitative patient preference assessment using large language models

**DOI:** 10.1371/journal.pdig.0001263

**Published:** 2026-03-12

**Authors:** Ted Grover, Emanuel Krebs, Deirdre Weymann, Morgan Ehman, Dean A. Regier

**Affiliations:** 1 Regulatory Science Lab, BC Cancer Research Institute, Vancouver, Canada; 2 Faculty of Health Sciences, Simon Fraser University, Burnaby, Canada; 3 School of Population and Public Health, University of British Columbia, Vancouver, Canada; Durham University, UNITED KINGDOM OF GREAT BRITAIN AND NORTHERN IRELAND

## Abstract

Patient experiences and perspectives are essential for shaping patient-centered healthcare. While large language models (LLMs) in healthcare are typically applied to specific clinical or patient-facing tasks, they have not been used for qualitative patient preference assessment, which often relies on thematic analysis to understand patient views expressed in interviews or focus groups. LLMs show initial promise for performing inductive thematic analysis of healthcare interview or focus group transcripts, yet no empirical studies have investigated LLMs to facilitate qualitative patient preference assessment. We employed the open-source Hermes-3-Llama-3.1-70B LLM to perform inductive thematic analysis on focus group transcripts from a previously published qualitative patient preference assessment study using three optimized prompt frameworks, and evaluated semantic similarity of LLM generated themes against human-analyzed themes using the Sentence-T5-XXL language embedding model. Sentence-level theme similarity was assessed using Jaccard similarity coefficients (0–1 range), computing coefficient scores across a broad range of discrete cosine similarity thresholds. We further evaluated LLM themes for similarity in lexical diversity and reading grade-level metrics and benchmarked semantic similarity results with published similarity thresholds previously used with qualitative healthcare data. All prompt frameworks generated themes with median Jaccard similarity coefficients with human-analyzed themes between 0.46–0.64, indicating moderate semantic overlap. Our best-performing framework instructed to pursue thematic saturation scored closest to human-analyzed themes on all reading grade-level metrics, and demonstrated 12% higher semantic overlap with human-analyzed themes compared to published benchmarks. Our worst-performing framework produced themes with moderate semantic overlap and hallucinated findings unidentified in human-analyzed themes. We demonstrate that LLMs can perform inductive thematic analysis of qualitative patient preference data, producing themes substantively similar in content and style to human-analyzed themes when augmented with sufficient domain-specific context. While LLMs may augment thematic analysis, the contextual nature of qualitative analysis remains a challenge requiring collaborative LLM frameworks integrating human expertise.

## Author summary

The experiences and preferences of patients provide valuable insights towards evaluating the risks and benefits of new health products, services, and technologies, and can help guide appropriate decision making along the development process. Patient interviews or focus groups are commonly used by researchers to develop a deep understanding of patient perspectives and the perceived benefits or risks of a new healthcare product, service, or technology. While this approach is effective, there is considerable manual effort and time required by researchers to uncover themes from the transcripts of these interviews or focus groups. In this study, we demonstrate that applying prompt optimization to open-source large language models can effectively and rapidly generate themes on patient preferences similar in content and style to human-analyzed themes. Our study can inform best practices for large language model use in thematic evidence generation of patient preferences to improve healthcare decision-making and accelerated patient-centered healthcare.

## Introduction

Patient experiences, perspectives and preferences are essential for shaping patient-centered healthcare and guiding priority setting in healthcare [1]. Clinicians, healthcare organizations and regulatory agencies continue to develop processes, guidelines, and initiatives to systematically integrate patient perspectives into decision making [1,2]. Patient preference assessment, which includes patient perspectives on the benefits and risks of new health products, services, and technologies, provides crucial evidence to support these efforts [3–6]. Generating patient preference evidence is resource intensive [1,2], presenting challenges for timely inclusion in decision-making processes [1].

Patient preference assessment uses qualitative research methods to develop a holistic understanding of patient perspectives on valued health and non-health outcomes, and perceived benefits and risks [1,2,7–9]. A common analytic method for qualitative patient preference assessment is thematic analysis [10]. Thematic analysis identifies meanings and patterns within written records of spoken language [10]. Approaches to thematic analysis exist on a continuum ranging from scientifically descriptive frameworks where themes are inductively generated, to interpretive frameworks that adopt more relativist and reflexive stances [11]. Thematic analysis requires iterative manual analysis and interpretation performed by researchers [11,12] with multiple researchers discussing and reaching agreement on coding and thematic categories [11,12]. Until recently, the complex and iterative nature of thematic analysis posed a barrier to AI-driven approaches.

Advancements in generative artificial intelligence present opportunities to accelerate thematic analysis and establish patient preference assessment research as a routine part of decision making. Large language models (LLMs), a type of generative artificial intelligence trained on unlabeled datasets through self-supervised learning, can understand and generate text with human-like coherence and fluency [13,14]. Most LLM applications in healthcare focus on specific clinical or patient-facing tasks [15,16] (e.g., clinical text summarization [17], medical question answering [18], medical diagnosis assistance [19], support cancer patients and their clinicians in decision making [20], and psychological assessment [21]) and are being rapidly integrated into clinicians’ daily workflows [22]. Prompt engineering methods such as self-reflection [23], iterative refinement [24], and step-by-step reasoning [25] can be combined into multi-step algorithms that significantly improve the quality and relevance of final LLM outputs [26,27]. These advancements facilitate explorations of LLM versus human labelled annotations of qualitative data across a diverse set of contexts and data sources [28]. To date, few empirical studies have focused on LLMs to promote and assist patient-centered healthcare, and none have specifically investigated applications of LLMs to assist or drive patient preference assessment.

Evidence designing and evaluating LLM-driven frameworks for inductive thematic analysis of qualitative healthcare data is sparse, with only two studies published to date. Mathis et al. [29] found that LLM-driven thematic analysis of transcripts from patient interviews in a psychiatric setting produced robust themes that passed face validity, and showed moderate semantic overlap with human-analyzed themes (Jaccard similarity coefficients of 0.44 -0.64 when using a relatively lenient binary cosine similarity threshold of 0.75) [29]. Drawing from this work, Raza et al. [30] employed efficient data processing and multiple prompting strategies to perform inductive thematic analysis of interview transcripts from parents of children with congenital heart disease. Using the same previously-used language embedding model with a more conservative cosine similarity threshold of 0.82, they reported a Jaccard similarity coefficient of 0.41 for their optimal framework, compared to 0.14 for the baseline framework described by Mathis et al. [29,30]. While these findings are promising, both studies used a relatively simplistic interpretation of Braun & Clarke’s (2006) six-step thematic analysis method to inform LLM framework structure and theme generation, with only minor variations in prompt instructions across studies [12,29,30]. Integrating qualitative theory into the prompt framework design, particularly in the initial open coding phase where a variety of different theoretical approaches exist, has not yet been thoroughly explored [31,32]. Both studies used an arbitrarily determined cosine similarity threshold for determining similarity, complicating the interpretation and contextualization of results across studies given potential sensitivity of the binary score to the chosen threshold [29,30]. Further, LLM generated themes have also yet to be directly compared to published peer-reviewed qualitative studies. More broadly, evaluating LLM themes against human-analyzed themes across other healthcare settings like patient preference assessment is needed to determine the generalizability, acceptability, and real-world applicability of LLM-driven thematic analysis.

We aimed to determine if an open-source LLM could be employed to effectively perform thematic analysis in a formal patient preference assessment context, and evaluate the degree to which different approaches to initial qualitative coding within prompt framework design could generate themes substantively similar in content and style to themes published in a prior peer-reviewed study. To achieve our study aims, we generalized the assessment of binary semantic similarity to be threshold independent, and evaluated the linguistic style of LLM themes through lexical diversity and reading grade level scores.

## Materials and methods

### Ethics statement

This study was approved by the University of British Columbia Research – BC Cancer Research Ethics Board (REB# H20-00861). Formal written consent was obtained by participants for their participation in the original focus groups study and the use of de-identified transcript data for secondary research.

### Study design

**Fig 1:**
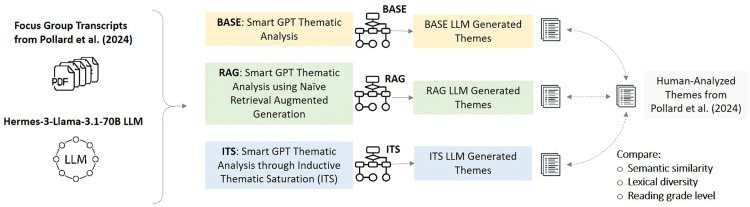
Overview of the study design and research procedures. We employ the Hermes-3-Llama-3.1-70B large language model (LLM) to perform inductive thematic analysis on five de-identified focus group transcripts from a prior qualitative patient preference assessment study [34]. We guide the LLM through three distinct prompt frameworks, resulting in three different sets of LLM generated themes. We evaluate these LLM themes against human-analyzed themes in terms of their semantic similarity (conceptual overlap), and similarity in lexical diversity and reading grade-level scores.

**Fig 2:**
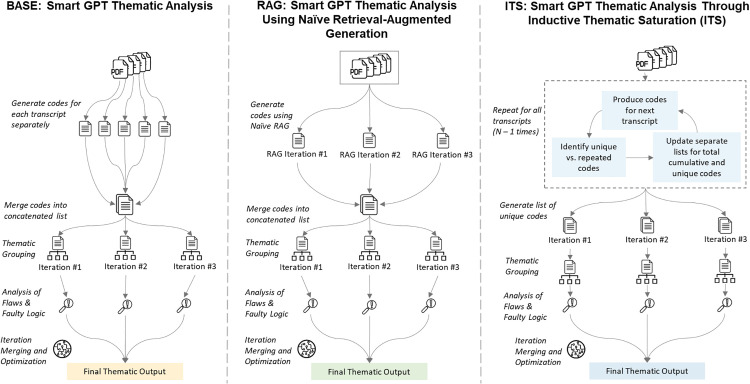
Procedural flowchart for LLM prompt frameworks. Arrows indicate transitions from between each distinct procedural step in the prompt framework.

Fig 1 presents an overview of our study design and research procedures. We employ the open-source Hermes-3-Llama-3.1-70B LLM [33] to perform inductive thematic analysis on de-identified focus group transcripts from a prior qualitative patient preference assessment study on patient perspectives toward health data-sharing platforms [34]. We guide the LLM through three distinct prompt frameworks (Fig 2), resulting in three different sets of LLM generated themes. Finally, we evaluate these LLM generated themes against human-analyzed themes in terms of their semantic similarity (conceptual overlap), and similarity in lexical diversity and reading grade-level scores.

### Data

The data we used were de-identified transcripts from a qualitative study by Pollard et al. [34]. This study investigated how secured health data-sharing platforms (SDSPs) could be established in a manner that acknowledges and responds to patient values, expectations, and concerns for enhanced precision oncology research [34]. Pollard et al. conducted five pan-Canadian focus groups between January 2022 and August 2023, recruiting N = 28 adults with a history of cancer to participate in health data sharing discussions [34]. Participants were 70% female, had a median age of 60 years, were 74% white, and 52% had a bachelor’s degree education or higher. Further details on participant demographics and characteristics are provided in Pollard et al. (Table 1, p.3) [34]. Two trained female qualitative researchers facilitated the English-language focus groups, while a trained male team member facilitated the single French-language discussion group [34]. Each focus group was approximately one hour in duration, and transcripts were professionally transcribed, translated (for the French focus group), de-identified and then validated against audio recordings [34]. Participants were de-identified by replacing all mentions of their name with a unique placeholder (e.g., ‘[PARTICIPANT 1]’). To mitigate against re-identification, keys linking original and de-identified transcripts to participant demographics were stored on a separate encrypted and password protected drive accessible only by the research team. Our five de-identified transcripts had a median word count of 10,097 (mean: 9,491). The range in word count across the five de-identified transcripts was 4,553 words (minimum: 6,534; maximum: 11,087). Further details on study eligibility and final participant characteristics are provided in Pollard et al. [34].

**Table 1:** Human-analyzed themes and LLM generated themes for each prompt framework.

Human-analyzed themes	BASE: LLM themes	RAG: LLM themes	ITS: LLM themes
**(1) Willingness to manage perceived risk**: Strategies to support enhanced data sharing while acknowledging the potential for data breaches, unintended secondary use of data, the development of cost-prohibitive treatments, and treatments with limited patient access.	**(1) Perceived benefits and motivations for data sharing:** Encompasses participant beliefs about how widespread sharing of health data can lead to improved cancer treatments and outcomes. They are motivated by the possibility of advancing medical research on a large scale and helping others through their contributions. Participants express enthusiasm for enabling new discoveries that could benefit future patients.	**(1) Data sharing motivations and willingness:** Encompasses participants’ willingness to share personal medical data for cancer research purposes and their motivations for doing so. It includes themes related to improving treatments, being involved in the process, and the value they place on data sharing for the greater good.	**(1) Data security & privacy concerns:** Encompasses participants’ concerns about protecting their personal health information from unauthorized access, breaches, and misuse when shared for research purposes. Participants express a desire for robust safeguards, secure platforms, and assurances that their data will be de-identified when shared. They have reservations about certain entities like pharmaceutical companies or insurance providers accessing sensitive information.
**(1.1) Oversight and regulation**: Established data-governance structures, mediation, and limiting access requests to only required data elements to support the proposed research, as mechanisms to enhance trust and mitigate risk perceptions. Risk mitigation strategies discussed included: review committees, patient involvement, and established processes for researchers requesting access to individual patient level data.	**(2) Privacy, security, and misuse concerns**: Many participants have significant reservations about the privacy, security, and potential misuse of personal health information. They worry about data breaches, cyberattacks, scams, unauthorized access by profit-driven entities like pharmaceutical companies or insurance providers. Ensuring anonymity is crucial for comfort in sharing data, along with having control over who can see their information and how it’s used.	**(2) Privacy and security concerns:** Participants express concerns about protecting personal information, ensuring anonymity, and controlling third-party access to shared medical data. They emphasize the need for technical security measures like encryption and oversight by trusted organizations.	**(2) Trust & oversight of data sharing:** Focuses on the importance of trustworthy organizations managing data sharing initiatives with transparency and patient representation in oversight roles. Participants want confidence that their information is handled responsibly by reputable entities they can trust, with mechanisms for accountability and transparency built into platform design.
**(1.2) Autonomy and connectedness**: Preservation of individual patient autonomy and identity within research initiatives, from data access to receipt of research outputs (e.g., treatment access). Notably, participants discussed flexibility in terms of what data could be shared (e.g., dynamic consent) and withdrawal of consent.	**(3) Oversight, trust, and transparency:** Building trust is essential through trusted gatekeepers controlling access to health information and legal safeguards against misuse. Participants have differing opinions on informed consent processes - whether it should be explicit or automatic within healthcare systems. Most want some level of control over their data.	**(3) Informed consent and transparency:** Relates to wanting clear communication about the consent process, understanding research outcomes, and receiving detailed explanations. Participants desire granular consent options rather than an ‘all or nothing’ approach.	**(3) Motivations & benefits of sharing:** This theme highlights the potential benefits of pooling health data for cancer research and what might motivate patients to contribute. Participants see value in advancing medical knowledge but also discuss possible personal incentives like compensation or access to research findings. Altruistic motivations are prominent.
**(1.3) Informational transparency**: Clear and transparent communication about research access and anticipated research outcomes as a means to ensure meaningful connectedness with research. Subtheme defined by variation in perspectives regarding the level of information that should be relayed to potential platform participants.	**(4) Public awareness and education:** There needs to be more awareness about the benefits of sharing health data beyond just cancer patients and education around research motivations. Transparency in how information will be used can address privacy concerns by informing participants upfront. Feedback on study results could incentivize participation further.	**(4) Compensation and incentives debate:** There is debate about offering monetary incentives for data sharing, with some feeling it could skew participation. Participants discuss honorarium payments for focus group involvement.	**(4) Patient autonomy & informed consent:** This theme relates to the importance of patient control and involvement in decisions about their health data, including granular consent options and diverse representation. Participants want a voice in how their information is used, clear guidelines on data usage, and assurance that all demographics are considered in research.
**(2) Navigating personal and population implications of secured data sharing:** Underlying value to support oncology research initiatives while mitigating the potential for harm and ensuring appropriate treatment of shared data.	**(5) Research inclusion and representation:** Participants want their data to contribute meaningfully, especially if it includes integrative oncology approaches. They advocate for diverse populations being included in research designs and receiving updates on findings that assure them of the value of their contributions.	**(5) Cross-jurisdictional collaboration:** Sharing data across provinces is seen as beneficial but participants question how selective sharing may affect research validity and want oversight of the process.	
**(2.1) Weighing risk and benefits**: Acknowledgment there is some personal risk involved (data hacking, data breaches, unintended access/use, discrimination), alongside the belief that potential benefit outweighs risks, if efforts are made to mitigate risks.			
**(2.2) Reciprocity**: Expectation and motivation to allow personal health data to be shared for the purposes of collective benefit of cancer patients (e.g., treatment development and better therapeutic alignment).			

Pollard et al. [34] concurrently analyzed the de-identified focus group data alongside data collection using a team of three analysts who conducted open coding and developed the coding framework using QSR NVivo Software [35]. The coding framework was iteratively developed by two of the analysts and then applied to subsequent transcripts [34]. Analytic themes were iteratively developed by collapsing and combining themes as the analysts coded each transcript [34]. Themes were finalized after analysts collectively determined no further focus groups were required based on no new perspectives being identified [34]. Final theme descriptions were finalized through member-checking with participants. Preferences for SDSP design and features were described separately from the set of final themes [34].

We compared LLM generated themes with verbatim theme summaries from Pollard et al. [34] as the set of ‘human-analyzed themes’ (see first column in Table 1). The major published themes were: (1) Willingness to manage perceived risk, and (2) Navigating personal and population implications of secured data sharing. The first theme had three minor themes: (1.1) Oversight and regulation; (1.2) Autonomy and connectedness; and (1.3) Informational transparency. The second theme had two minor themes: (2.1) Weighing risk and benefits; and (2.2) Reciprocity [34]. We excluded LLM themes related to SDSP design elements as these were described in length separately and not included in the analytic themes table (p.5) in Pollard et al. [34]. Table A in S1 File Supporting Information presents themes related to SDSPs that were excluded from both our set of human-analyzed and LLM generated themes.

### LLM selection and deployment

We locally employed the Hermes-3-Llama-3.1-70B (Hermes-3) LLM [33], a specialized version of Meta’s open-source Llama-3.1-70B LLM [36]. Hermes-3 was trained by its developers to encourage following both system and instruction prompts exactly and neutrally [33]. Our choice of LLM was informed by our desire to demonstrate how LLM-driven approaches to patient preference assessment could be made accessible to researchers operating in settings similar to our own where compute resources are limited, and data privacy constraints require that LLMs are open-source and locally deployed. 70B parameter LLMs were the largest and most powerful open-source models that could be realistically employed given these considerations. Specific procedures on how we chose Hermes-3 versus similarly sized open-source LLMs is provided in Text A in S1 Supporting Information (S1 File).

We selected hyperparameters for Hermes-3 through experimentation to balance consistency, variability, and contextual insight of the LLM’s output for our study context. The first key criterion for determining LLM hyperparameters was that the LLM should produce slight variations in its response, both in terms of word choice and semantic representation (e.g., how it chose to merge codes into themes) for different iterations of the same prompt. Second, the hyperparameters should maintain constituent linguistic style and formatting across varied responses. These criteria enabled the iterative refinement and reflection techniques within our prompt frameworks to be most effective.

Hyperparameters tested included Temperature, Top P Sampling, Min P Sampling, Repeat Penalty, and Top K Sampling. Temperature controls the randomness in the model’s predictions, with higher values leading to more creativity and variance, and lower values producing more conservative responses. Top P Sampling acts similarly to temperature, and represents the minimum cumulative for the possible next tokens chosen in the output. Min P Sampling is the minimum base probability for a token to be selected for output. Repeat penalty is the amount the model is discouraged from repeating the same token in its output. Top K Sampling limits the next output token to one of the most top-k probable tokens. We iteratively tested values ranging from.80 to 1 by increments of.10 for Temperature,.05 to.25 by increments of.05 for Top P Sampling.01 to.05 by increments of.01 for Min P Sampling, and 1 to 1.3 by increments of.05 for Repeat Penalty, and 25–45 by increments of 5 for Top K Sampling. The final hyperparameter settings we deployed were: Temperature: 1; Top P Sampling: 0.15; Min P Sampling: 0.02; Repeat Penalty: 1.15; Top K Sampling: 40.

We deployed Hermes-3 on a local Windows machine equipped with a NVIDIA GeForce RTX 4070 GPU and 64GB of system RAM. We used a 4-bit quantization of the model that allowed for an optimized model size that fit within our system RAM limits, without a significant loss in performance relative to the non-quantized version [37]. We used a context window size of 34,500 for Hermes-3 which allowed the longest focus group transcript to be passed entirely to the LLM during prompting steps.

### LM studio

We used a GGUF model format of Hermes-3 [38], and conducted all analysis and inference with the model through LM Studio [39]. LM Studio is a desktop application for running local LLMs, with a graphical user interface (GUI) for interacting and saving all conversation histories with LLMs [39]. LM Studio operates entirely offline, ensuring privacy and security for personal health information, and providing a system to manage local models and their fine-tuned configurations [39].

### LLM prompt frameworks

In the context of our study, we define SmartGPT as the systematic layering and integration of prompt engineering techniques like self-reflection [23], iterative refinement [24], chain-of-thought reasoning [25], and LLM-as-judge evaluation [40] into prompt framework design. We evaluated three different frameworks for generating LLM themes using Hermes-3 (Fig 2): (1) BASE: SmartGPT Thematic Analysis; (2) RAG: SmartGPT Thematic Analysis Using Naïve Retrieval-Augmented Generation; and (3) ITS: SmartGPT Thematic Analysis through Inductive Thematic Saturation. All frameworks employed the same broad SmartGPT prompting approach, used the same model hyperparameters, and the same prompt text for initial transcript analysis, thematic grouping, analysis of flaws and faulty logic, and final theme merging and optimization (see Fig B, Fig C, and Fig D in S1 Supporting Information (S1 File)). We employed zero-shot prompting, where prompts included detailed information on the task and expected output [41]. Prior work found that zero-shot prompting produced LLM themes with the best semantic overlap with human themes compared to alternative one-shot prompting where prompts include an example to guide LLM output [30]. Framework prompts for final theme merging and optimization did not include any explicit instructions to group themes together nor put any limitations on word count in its final output in an effort to be impartial to the structure and format of our human-analyzed themes. The key procedural difference across frameworks was the approach taken to generate and format the lists of qualitative codes used in subsequent thematic grouping steps. While the descriptions below for code generation steps use the word ‘code(s)’, this term has specific meaning within thematic analysis that sometimes confused our LLM. We used the term “theme” instead throughout when prompting the LLM. Fig B, Fig C, and Fig D in S1 Supporting Information (S1 File) provide the prompt text used in each procedural step (where applicable), for BASE, RAG, and ITS respectively.

### System prompt

Before executing each framework, we first initialized a system prompt for the LLM that set the general context for the LLM’s expected role as an academic researcher and expected guidelines for its responses: “*You are an academic researcher tasked with performing thematic analysis of qualitative data. The answers you provide to all user prompts should be detailed, thorough, and use a scholarly tone of writing*.” This system prompt was the same for all subsequent tasks prompts provided to the LLM across all three frameworks.

#### BASE: SmartGPT thematic analysis.

BASE followed Mathis et al. [29] but with more detail in prompts on the study data and expectations for LLM responses. BASE contained five steps for theme generation (see Fig B in S1 Supporting Information (S1 File)):

i. The LLM identified all codes from each transcript individually, resulting in a list of five separate lists of codes.
ii. All five lists of codes were concatenated into a new document (including duplicate or very similar codes).
iii. The LLM determined how the list of concatenated codes could be grouped together into thematic groups. This step was iterated three separate times.
iv. For each separate thematic grouping iteration, the LLM evaluated the thematic grouping it provided for any flaws or faulty logic.
v. Each thematic grouping and associated analysis of flaws or faulty logic were transferred to three separate documents. The LLM then determined the best possible set of final themes from these three documents.

Fig F and Fig G in S1 Supporting Information (S1 File) show example prompt input and LLM output for steps iii. and iv. in BASE respectively.

#### RAG: SmartGPT thematic analysis using naïve retrieval-augmented generation.

RAG provided all five focus group transcripts together to the LLM and leveraged naïve retrieval-augmented generation [42] to generate initial codes. Naïve retrieval-augmented generation enables LLMs to more efficiently respond to user prompts with reference to a set of documents by dividing the set of documents into smaller chunks, then retrieving chunks most similar to the user prompt, before augmenting the LLMs response using these retrieved chunks as context [42]. RAG provides a less CPU-intensive and more time-efficient method for the LLM to analyze and generate initial codes from all focus group transcripts in a single prompt, rather than each transcript being analyzed independently. This approach relies, in part, on the LLM’s existing knowledge and training data in the specialized domain of the case study data to retrieve the information most relevant to the prompt, and is more prone to reflect any biases present in this existing knowledge [42]. Compared to advanced retrieval approaches, naïve retrieval-augmented generation approaches are known to sometimes struggle with precision and recall during the retrieval phase, especially when processing complex or multi-step prompts, leading to coherence issues in generated responses [42].

Naïve retrieval-augmented generation and transcript chunking were triggered automatically in LM Studio as our maximum context size (i.e., token count) window was less than the combined token count of all five transcripts. We iterate the first coding step using naïve retrieval-augmented generation three separate times, resulting in three separate lists of codes. These three lists were then concatenated into a new document. Finally, RAG followed the same procedure and prompt language as steps iii, iv, and v in BASE to generate final themes (Fig C in S1 Supporting Information (S1 File)).

#### ITS: SmartGPT thematic analysis through inductive thematic saturation.

ITS employed the concept of inductive thematic saturation within the initial code generation step [31,32]. The concept of saturation, broadly defined as a criterion for determining when data collection and/or analysis can be stopped [31], motivated this approach. The application of inductive thematic saturation in the context of LLM-driven thematic analysis is described by De Paoli & Mathis [32], where the ratio of unique codes to total codes identified after all transcripts have been analyzed acts as a synthetic indicator of how much inductive thematic saturation the LLM has achieved, i.e., Inductive Saturation Ratio = Unique Codes/ Total Codes. As additional transcripts are each analyzed by the LLM, the number of new codes identified by the LLM, and the ratio of unique codes to total codes across all transcripts analyzed should decrease as more thematic saturation is achieved [32].

The first five transcripts were randomized in order, and the LLM identified all codes from the first transcript. These first codes were then added to both the list of unique codes and total codes. For each next transcript from the randomized order, the LLM again identified all codes, then identified which codes were repeated (i.e., convey the same idea or meaning) from the set of unique codes. The lists of total codes and unique codes were then updated. This process was iterated two additional times, resulting in three separate sets of unique codes. Each set of codes is independently run through the same procedure and prompt language as steps iii, iv in BASE before being combined into a set of final themes using the same procedure and prompt language as step v in BASE (Fig D in S1 Supporting Information (S1 File)). Fig E in S1 Supporting Information (S1 File) presents a plot of the ITS ratio using the ITS framework across the five transcripts.

### Semantic similarity

We used the Sentence-T5-XXL language embedding model to quantitatively measure the semantic similarity between human-analyzed and LLM generated themes [43]. The Sentence-T5-XXL model is specifically designed and optimized to excel at sentence similarity tasks, and is one of the most robust models of its type currently available [43]. This model first encodes text into 768-dimensional vectors, providing a detailed and nuanced representation of sentence semantics. The similarity of two sentences is then computed using the cosine similarity of the sentences vector representations, resulting in a similarity score between 0 and 1, with higher scores indicating stronger similarity and 1 representing identical similarity [43].

For each prompt framework, we apply the Sentence-T5-XXL model to all possible pairs of LLM generated vs. human-analyzed pairs. As the sample of resulting cosine similarity scores is relatively small (N <= 35) for each framework, we used non-parametric tests to compare across frameworks. We used two-tailed Kruskal-Wallis and Dunn tests to examine statistically significant differences in median cosine similarity scores between frameworks, with Benjamini-Hochberg adjusted p-values for multiple comparisons [44].

We computed binary similarity scores across a broad range of discrete cosine similarity thresholds to improve on prior work by providing a threshold-independent characterization of semantic similarity, and evaluated whether the score distributions of each prompt framework differed significantly. We calculated the Jaccard similarity coefficient as the proportion of human-analyzed vs. LLM generated theme pairs reaching or exceeding our similarity threshold, and hit rate as the proportion of human-analyzed themes that reach or exceed our similarity threshold with at least one LLM generated theme. Higher Jaccard coefficients indicate a greater proportion of similar human-LLM theme pairs, while higher hit rates indicate that more human themes were adequately captured by LLM themes. As past work using the sentence-t5-XXL embedding model has used binary cosine thresholds of 0.75, 0.775, and 0.82 [29,30], we used 0.80 as a conservative median threshold and computed both metrics across a similarity threshold range of 0.70 to 0.90 by 0.01 increments using the sentence-t5-XXL model. We then used two-tailed Kruskal-Wallis and Dunn tests to examine statistically significant differences in median Jaccard similarity coefficients and hit rate between frameworks, with Benjamini-Hochberg adjusted p-values for multiple comparisons [44].

To benchmark our results with past work that used a single binary cosine similarity threshold, we also reported our Jaccard similarity coefficient and hit rate obtained using the threshold of 0.82 employed by Raza et al. using the same sentence-t5-XXL model [30]. As an internal benchmark, we also computed cosine similarity scores between all LLM framework theme sets (e.g., BASE vs. RAG), and corresponding Jaccard similarity coefficients using the same benchmark threshold of 0.82 (Fig A in S1 Supporting Information (S1 File)).

### Lexical diversity and reading grade level

To measure language complexity or richness (i.e., lexical diversity) we computed HD-D (Hypergeometric Distribution Diversity) [45] and MATTR (Moving Average Type Token Ratio) [46] on the concatenated theme descriptions for both the human-analyzed and LLM generated theme sets respectively. Higher MATTR and HD-D scores indicate greater lexical diversity within a given text. MATTR and HD-D indices have been shown to accurately reflect the lexical diversity of a text regardless of its length [47].

We also computed the Coleman-Liau Index (CLI) [48], Flesch-Kincaid Grade Level (FK) [49], and Gunning Fog Index (FOG) [50] readability metrics on the same concatenated theme descriptions as a proxy to determine if LLM themes reached or exceeded readability standards of most academic writing. We estimated a global lower bound threshold score for academic level writing across all three metrics based on prior literature. A past analysis of 3.3 million academic abstracts from PubMed.org reported a mean CLI score of 16.86 [51]. Scores of 15 for FK and 17 for FOG are reported as the typical lower bound of most academic papers [49,50]. We therefore used a readability score of 17 across all three metrics as the conservative lower bound threshold score for the reading grade level of typical academic writing.

## Results

Table 1 presents the final human-analyzed and LLM themes generated by each of the prompt frameworks: BASE, RAG, and ITS. All prompt frameworks had notable similarities, generating themes broadly focused on: i) the perceived benefits and motivations for data sharing, ii) privacy and security concerns, and iii) oversight and transparency. Overall, we observed moderate to strong semantic similarity between LLM vs human-analyzed themes, and LLM generated theme sets achieved similar lexical diversity and reading grade level scores compared to our reference human-analyzed themes.

### Semantic similarity of LLM generated themes

**Fig 3:**
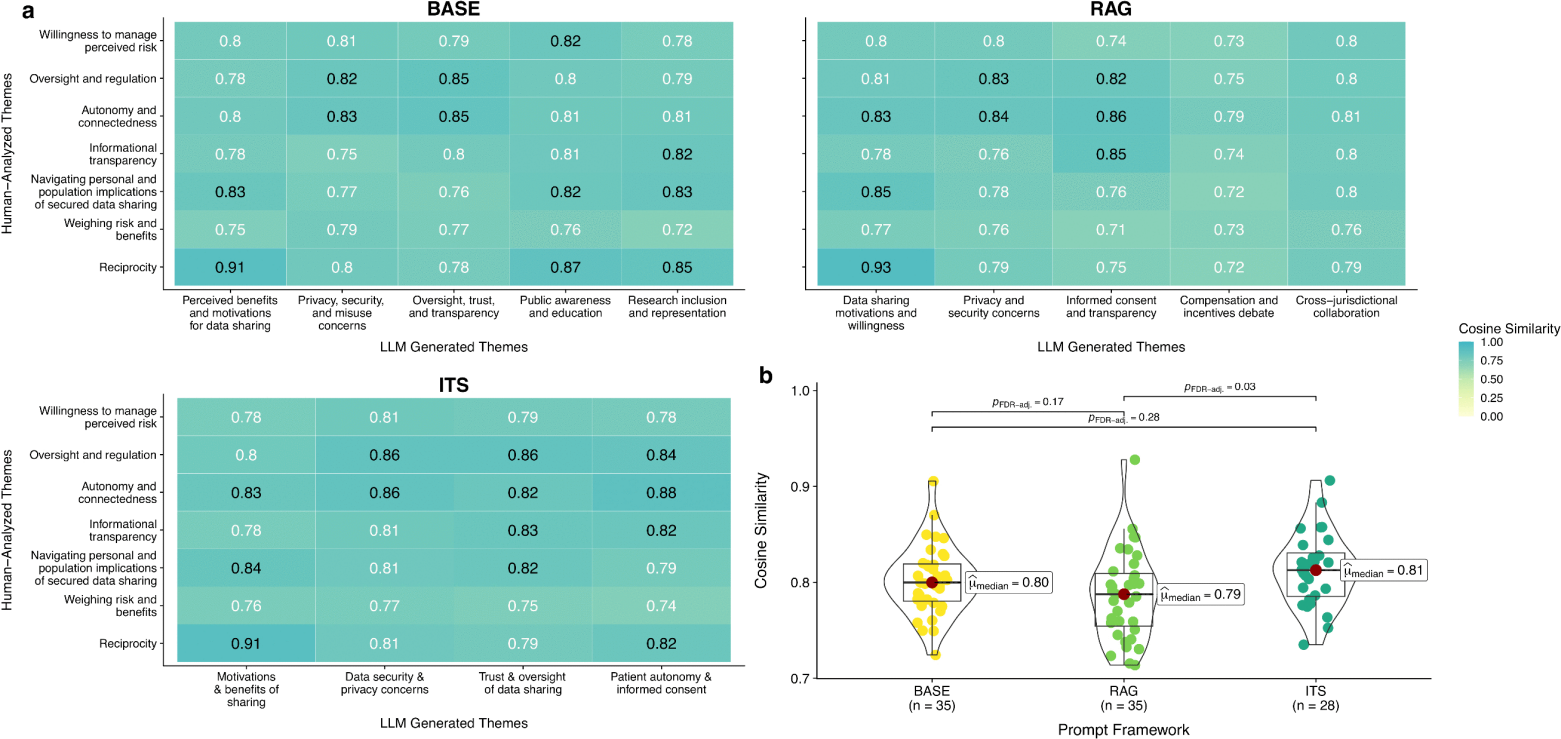
Semantic similarity of LLM generated themes. **a)** Cosine Similarity heat plot for the Sentence-t5-XXL model. The numbers in the tiles represent the cosine similarity scores between LLM generated themes (x-axis) and human-analyzed themes (y-axis) description pairs. Black text signals a cosine similarity meeting a binary similarity threshold of 0.82. **b)** Violin box plot and statistical tests for differences in median Sentence-t5-XXL computed cosine similarity vs. human-analyzed themes across the three frameworks. P-values for pairwise comparisons between frameworks are controlled for false discovery rate (FDR) using the Benjamini-Hochberg procedure.

Fig 3 presents the semantic similarity scores between human-analyzed and LLM generated themes for each prompt framework, with median cosine similarity with human-analyzed themes of 0.80 (BASE), 0.79 (RAG), and 0.81 (ITS) respectively. These scores indicate strong conceptual similarity between human and LLM generated themes. Across the LLM prompt frameworks, we observed a maximum and minimum cosine similarity of 0.93 and 0.71 respectively between human-analyzed vs. LLM generated theme pairs, indicating heterogeneity in conceptual similarity between themes. The Kruskal-Wallis test indicated a statistically significant difference in the median cosine similarity with human-analyzed themes between the three LLM prompt frameworks (X^2^_Kruskal-Wallis_ (2) = 6.79, *P* = .03, CI_95%_ [0.01, 1.00]), with a statistically significant pairwise difference between the median cosine similarity of RAG vs. ITS (*P*_FDR-adj_ = 0.03). There was no significant pairwise difference in median cosine similarity between BASE vs. RAG (*P*_FDR-adj_ = 0.17), nor between BASE vs. ITS (*P*_FDR-adj_ = 0.28).

**Fig 4:**
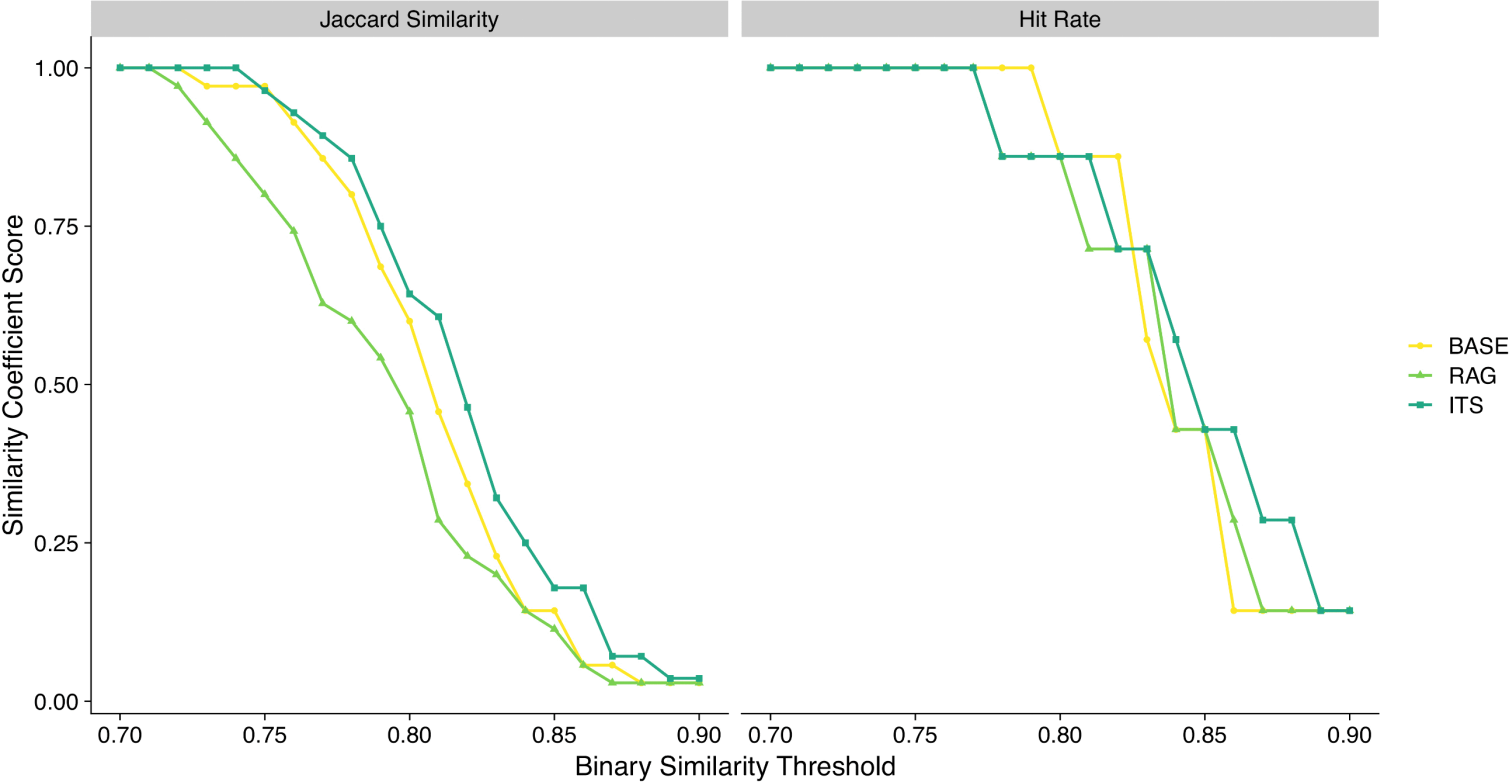
Jaccard similarity coefficients and hit rates for LLM generated themes. Jaccard Similarity coefficients (left) and hit rate scores (right) plotted for each of the 21 binary similarity thresholds tested, for each prompt framework.

Fig 4 presents plots for computed Jaccard similarity coefficients and hit rates across the range of cosine similarity thresholds tested. Median Jaccard similarity coefficients were 0.60 (BASE), 0.46 (RAG), and 0.64 (ITS) respectively, indicating moderate median thematic congruence or overlap between human-analyzed and LLM generated themes across frameworks. While ITS achieved a higher Jaccard similarity score compared to BASE and RAG for 17 of the 21 similarity thresholds computed, the Kruskal-Wallis test indicated no statistically significant difference in median Jaccard similarity coefficients between the three prompt frameworks (X^2^_Kruskal-Wallis_ (2) = 1.79, *P* = .41, CI_95%_ [0.004, 1.00]). Median hit rates were 0.86 (BASE), 0.86 (RAG), and 0.86 (ITS) respectively, with no statistically significant difference in median hit rate between the three prompt frameworks (X^2^_Kruskal-Wallis_ (2) = 0.14, *P* = .93, CI_95%_ [0.001, 1.00]).

Comparing our results to prior work using the similarity threshold from Raza et al. of 0.82, the Jaccard similarity coefficients were 0.34 (BASE), 0.23 (RAG), and 0.46 (ITS), with ITS exceeding the best semantic similarity reported by Raza et al. of 0.41. Comparing Jaccard similarity scores between all LLM theme sets using this similarity threshold resulted in coefficients of 0.32 (BASE vs. RAG), 0.75 (BASE vs. ITS), and 0.60 (RAG vs. ITS), indicating LLM generated theme sets generally had more thematic congruence with each other than with human-analyzed themes (Fig A in S1 Supporting Information (S1 File)). Using the same similarity threshold of 0.82, the hit rates were 0.86 for BASE, 0.71 for RAG, and 0.71 for ITS. The human-analyzed theme ‘Weighing risk and benefits’ did not meet or exceed either cosine similarity threshold with any LLM generated theme, reaching a maximum of 0.79 for BASE. RAG generated a final theme unidentified in human-analyzed themes entitled ‘Compensation and incentives debate’ that was related to participant concerns about honorarium payments for focus group participation; information that is not pertinent to the research focus of the original study [34].

### Lexical diversity and reading grade level of LLM generated themes

**Fig 5:**
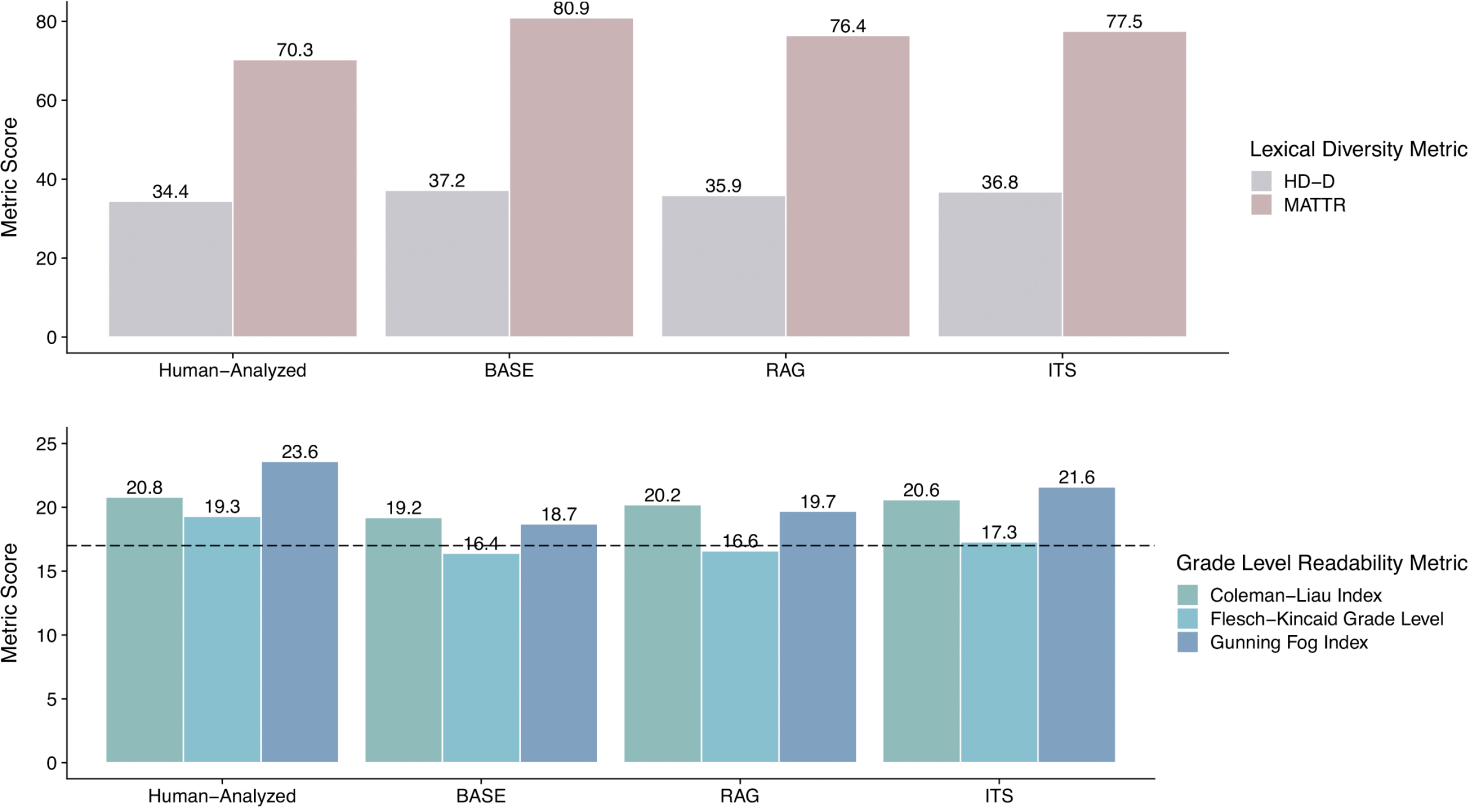
Lexical diversity and reading grade level scores. **a)** Lexical diversity scores for human-analyzed and LLM generated framework theme sets respectively. MATTR scores are multiplied by 100 for easier visualization. **b)** Grade level Readability Index scores for human and LLM framework theme sets respectively. The horizontal dotted line references a readability score of 17, indicating a conservative lower bound threshold for the average grade level score for most academic writing.

Fig 5 presents the lexical diversity (i.e., richness) and reading grade level scores for both human-analyzed and LLM generated theme sets. Overall, across prompt frameworks, the final LLM generated theme sets achieved similar lexical diversity and reading grade level scores compared to our reference human themes.

BASE, RAG, and ITS theme sets scored slightly higher than human themes for both the HD-D (Human-analyzed: 34.4, BASE: 37.2, RAG: 35.9, and ITS: 36.8) and MATTR (Human-analyzed: 0.70, BASE: 0.81, RAG: 0.76, and ITS: 0.78) lexical diversity metrics. In contrast, the human-analyzed themes scored slightly higher versus BASE, RAG, and ITS themes for all grade-level readability metrics calculated (CLI, FK, and FOG). All LLM theme sets exceeded the lower bound score of 17 on both CLI and FOG, indicating that LLM generated theme descriptions roughly matched the post-graduate reading level of most academic writing. ITS scored highest versus other prompt frameworks, and scored closest to human-analyzed themes, for all readability metrics (ITS; CLI: 20.6, FK: 17.3, FOG: 21.6, Human-analyzed; CLI: 20.8, FK: 19.3, FOG: 23.6).

## Discussion

This study demonstrates that an LLM can effectively conduct inductive thematic analysis in a qualitative preference assessment study context. By establishing how LLMs can rapidly generate themes similar in content and style to those published in peer-reviewed work, we present a practical AI-driven approach to the conduct of qualitative patient preference assessment and provide insights into the strengths and limitations of different prompting strategies. Our approach to determining binary semantic similarity across a range of thresholds also provides a more generalizable interpretation and contextualization of similarity results. While all prompt frameworks were able to generate themes with moderate overlap to previously published themes, our thematic analysis framework using inductive thematic saturation (ITS) produced themes that best mirrored both the thematic content and academic writing style of our human-analyzed themes. In contrast, our framework using naïve retrieval-augmented generation (RAG) produced themes with the least semantic overlap with human themes and hallucinated a theme unidentified in human-analyzed themes.

Our framework with inductive thematic saturation (ITS) achieved an improved semantic overlap between LLM themes and human themes compared to all previously published benchmarks using qualitative healthcare data. Prior work used the same embedding model and method for quantifying binary semantic similarity, and our ITS framework achieved 12% higher binary semantic similarity compared to the best reported framework by Raza et al. [30], which outperformed Mathis et al. [29]. Our threshold-independent approach to scoring binary semantic similarity allowed us to better contextualize these results. We found that while ITS had the highest Jaccard similarity coefficients across our three frameworks for a large majority of similarity thresholds tested, including the 0.82 threshold used by Raza et al. [30], differences in median Jaccard similarity coefficient between the three frameworks were not statistically significant. Our increased level of detail in zero-shot prompts, inclusion of a context setting system prompt, integration of a specific approach to thematic saturation in the initial coding stage, use of multiple coding iterations, and deploying an LLM fine-tuned to follow instructions precisely and neutrally, all potentially contributed in part to improvements in threshold-dependent semantic overlap with human themes observed. In contrast, our RAG framework using naïve retrieval-augmented generation was the only prompt framework to generate a final theme unrelated to the research context. All themes generated by BASE and ITS frameworks had surface-level coherence and clear relation to the research goals. This hallucinated theme observed in our RAG framework can be categorized as context inconsistency hallucination, where the LLM fails to adhere to contextual information provided by the user [52,53]. These findings highlight the limitations of existing open-source LLM knowledge and the potential for context inconsistency hallucinations when applying naïve augmented retrieval approaches, particularly in specialized domains [53].

LLM-driven thematic analysis can facilitate the rapid generation of evidence and reduce burden placed on human analysts. It took approximately 3.5 hours for a single analyst to execute our ITS framework on a collection of transcripts totaling nearly 50,000 words using a workstation with modest computational power and available memory (RAM). LLM-driven thematic analysis could more rapidly generate thematic evidence informing choice task development and framing, or attribute selection decisions for quantifying patients’ preferences and risk-benefit trade-offs [3,4,8]. Reduced manual effort requirements could also expand research capacity, enabling more time-sensitive patient preference assessment studies to be conducted in parallel. Finally, LLM-driven thematic analysis frameworks could facilitate improved methodological consistency and standardization in real-world thematic evidence generation, addressing some barriers to further integration of patient preference assessment into regulatory and reimbursement decisions [1].

Evaluation and integration of LLMs for patient preference assessment faces unique challenges as qualitative analysis relies on interpretive, open-ended judgements rather than objectively verifiable outcomes. Prior work has shown that LLMs can outperform human experts in predicting novel outcomes from experiments on the basis of past data [54]. While both scientific outcome prediction and qualitative analysis are forward-looking contexts where the future outcome is initially unknown, scientific outcome prediction always requires clear, objective criteria for determining the correctness of a generated prediction [17,18,54]. In contrast, inductive thematic analysis relies on the subjective interpretation of qualitative researchers, and multiple analysts may derive different, yet equally valid interpretations from the same dataset [7,11]. As a result, extending conclusions about LLM superiority from predictive, closed-form tasks to interpretive and open-ended contexts like qualitative analysis raises epistemological challenges regarding what constitutes ‘accuracy’ in qualitative research. In this study, we compared LLM generated themes to previously published human-analyzed themes as a pragmatic starting point to demonstrate their surface-level coherence, and the notable effect that prompt framework design can have on final theme quality. However, such comparisons should not be seen as tests of analytic validity. These challenges highlight the need to develop evaluation frameworks for LLM-driven TA that draw on principles more appropriate to interpretive analysis. Finlay [11] proposes the ‘4 R’s’ (rigour, relevance, resonance, and reflexivity) as guiding criteria for assessing qualitative analysis. Even once evaluation criteria are established, there remains difficult regulatory and ethical decisions regarding the extent that LLM generated thematic evidence can or should influence decision-making processes.

As generative AI becomes integrated into qualitative workflows, researchers, regulatory agencies, and health technology assessment (HTA) organizations must establish guidance specific to LLM use in patient preference assessment. While new regulatory category and safeguarding initiatives exist to mitigate potential harms, maintain ethical standards, and protect patient privacy when using LLMs in clinical settings, comparable initiatives are lacking for patient preference assessment [55,56]. Compared to use in clinical contexts, LLM use in patient preference assessment requires distinctly different characterizations of concepts like ‘harm mitigation’ and ‘efficacy’, particularly for qualitative study contexts where no ground truth data exists. Given the potential of generative AI to transform regulatory and HTA decision making, there is a pressing need for guidance to balance accelerated innovation with scientific rigor and health equity considerations [57–60]. Our work provides empirical evidence for a specific use case of LLMs in patient preference assessment and can inform guidance specific to LLM use in this context to improve healthcare decision-making.

There are limitations to our study. First, our work used only one source of qualitative healthcare data and associated human-analyzed themes. While these data were high quality and published in a peer-reviewed academic journal [34], generalizability of our approach has yet to be established. The impact of additional data on LLM-derived themes is uncertain, highlighting an area for further research. Second, we evaluated just one open-source LLM as we aimed to provide an initial case study of LLM-driven TA in a patient preference assessment context. Future work should investigate relative performance in a similar context across multiple LLMs varying in their pre-training approaches and training parameter sizes. Third, unlike past work that also included a human evaluation of thematic similarity, we employed a single language embedding model method to compare semantic similarity [29,30]. We chose against conducting a human-evaluation of similarity as our study aimed to demonstrate an accessible and practical LLM-driven approach to patient preference assessment that minimized manual human effort in both the generation and evaluation of LLM themes. Our more robust and threshold-independent approach to assess binary similarity also addressed prior limitations that necessitated more additional human evaluation to validate semantic similarity computed using a single predetermined cosine threshold [29,30]. Finally, no LLM generated theme met the similarity threshold for the human-analyzed theme on weighing risks and benefits of secured health data-sharing platforms. While we intentionally designed our prompts to be as impartial as possible to our human-analyzed themes to avoid confounding our similarity evaluations, the use of in-context learning [41] and fine-tuning procedures [61] to better adapt open-source LLMs to the norms of patient preference research could help address the limitations of our blinded approach.

There also exist broader limitations and potential barriers for others adopting our approach in real-world settings. LLMs have been observed to sometimes perpetuate long standing biases in healthcare (e.g., around sex, gender, or race) [58,62]. Transparency in researcher assumptions and analytic process is a requirement of good thematic analysis [11], and LLM-driven thematic analysis should aim to adhere by these requirements as well. Some recently developed open-source LLMs now enable transparency into chain-of-thought (CoT) reasoning [63] and decision-making steps informing each LLM response, which could assist the identification of potential biases in LLM reasoning and outputs [63]. Future work should also explore other bias mitigating methods such as member-checking LLM generated themes with study participants, or employing human domain expert review of LLM generated themes [30,64]. Finally, all studies that employ LLMs with multi-step prompt frameworks may face reproducibility challenges due to the inherent non-deterministic nature by which LLMs generate their outputs [65]. While variability in output across iterations of the same prompt is optimal for techniques like iterative refinement and LLM-as-judge evaluation [24,40], optimizing model hyperparameters for this behavior may make traditional comparative design and evaluation of these techniques more difficult in practice [65].

### Conclusion

We demonstrate that LLMs can perform inductive thematic analysis in a patient preference assessment study context, producing themes substantially similar in content and style to human-analyzed themes. Our findings highlight the potential of LLMs to support patient preference assessment through rapid evidence generation and to facilitate its integration into routine healthcare decision making. At the same time, evaluation and eventual integration of LLM-driven thematic analysis for patient preference assessment is complicated by the open-ended nature of qualitative analysis and systematic biases that LLMs can contain. As research in this novel domain flourishes, both researchers and government agencies should increase collaborative efforts to develop best practices for LLM use in qualitative patient preference assessment.

## Supporting information

S1 File Supporting information: Additional supporting figures, tables, and text. (DOCX)
