# Supplementary material for: Advancing the science of qualitative patient preference assessment using large language models
Source: PLOS Digit Health. 2026 Mar 12;5(3):e0001263. doi: 10.1371/journal.pdig.0001263 (PMC12981457; doi:10.1371/journal.pdig.0001263)
Supplement: S1 File — (DOCX) [file pdig.0001263.s001.docx]

**S1 Supporting Information.**

**Advancing the Science of Qualitative Patient Preference Assessment using Large Language Models**

Ted Grover, PhD^1^, Emanuel Krebs, MA^1^, Deirdre Weymann, MA^1,2^, Morgan Ehman, MPH^1^, Dean A. Regier, PhD^1,3,*^

1. Regulatory Science Lab, BC Cancer Research Institute, Vancouver, Canada.

2. Faculty of Health Sciences, Simon Fraser University, Burnaby, Canada

3. School of Population and Public Health, University of British Columbia, Vancouver, Canada.

**Table A. Themes related to secure data sharing platforms (SDSPs) excluded from both human-analyzed and LLM generated themes.**

| **Human-Analyzed SDSP Theme(s)** | **Framework A: LLM SDSP Theme(s)** | **Framework B: LLM SDSP Theme(s)** | **Framework C: LLM SDSP Theme(s)** |
| --- | --- | --- | --- |
| **Platform Features**: Cutting across themes, participants discussed granular aspects of SDSP design, interface, and optional features facilitating greater connectedness with the research and study findings. Notably, participants stressed the importance of ensuring the platform interface meets the needs of those with varying degrees of risk tolerance, informational curiosity, and engagement with the research process and outputs. | **Platform Design Preferences:** Participants desire a user-friendly platform that allows granular consent options and clear communication about data usage. They want strong security measures like anonymization of personal details. Involving patients in the design process can improve credibility and usability for diverse populations. | **Platform Design Preferences:** Participants provide suggestions for designing an intuitive, user-friendly data sharing platform with accessible language, visuals, and simplicity. They discuss preferred communication methods. | **Platform Design & User Experience:** This group encompasses themes related to the design of user-friendly platforms that simplify secure data sharing while respecting privacy. Participants desire intuitive systems with straightforward consent processes and protections built into the architecture. |

**Text A. Procedures for choosing Hermes-3 versus similarly sized open-source LLMs.**

70B parameter open-source LLMs with 4-bit quantization were the most powerful models that could be run given our hardware limitations, and in the research settings of prior work. At the time of our study in January available open-source 70B LLMs were Hermes-3-Llama-3.1-70B (Hermes-3), Qwen2.5-72B-Instruct (Qwen-2.5), Llama-3.1-70B-Instruct (Llama-3.1). We auditioned each 70B LLM using its respective developer recommended default hyperparameters, evaluating it on our most complex ITS framework based on completion time and frequency of prompt interpretation errors. Hermes-3 was able to complete ITS in approximately 3.5 hours without any noted interpretation errors. In contrast, both Llama-3.1 and Qwen-2.5 completed ITS in approximately 4 hours, and did not consistently follow instructions to identify repeated codes gathered from previous coding iterations. These findings motivated the use of Hermes-3 for our study context.

**Fig A. Semantic similarity between LLM generated themes.** a) Cosine Similarity heat plot for Sentence-t5-XXL model computed cosine similarity between all LLM generated theme sets. Faded text color signals a cosine <= 0.82. b) Jaccard similarity coefficients for each LLM generated theme framework comparison computed using a binary cosine similarity threshold of 0.82.

**
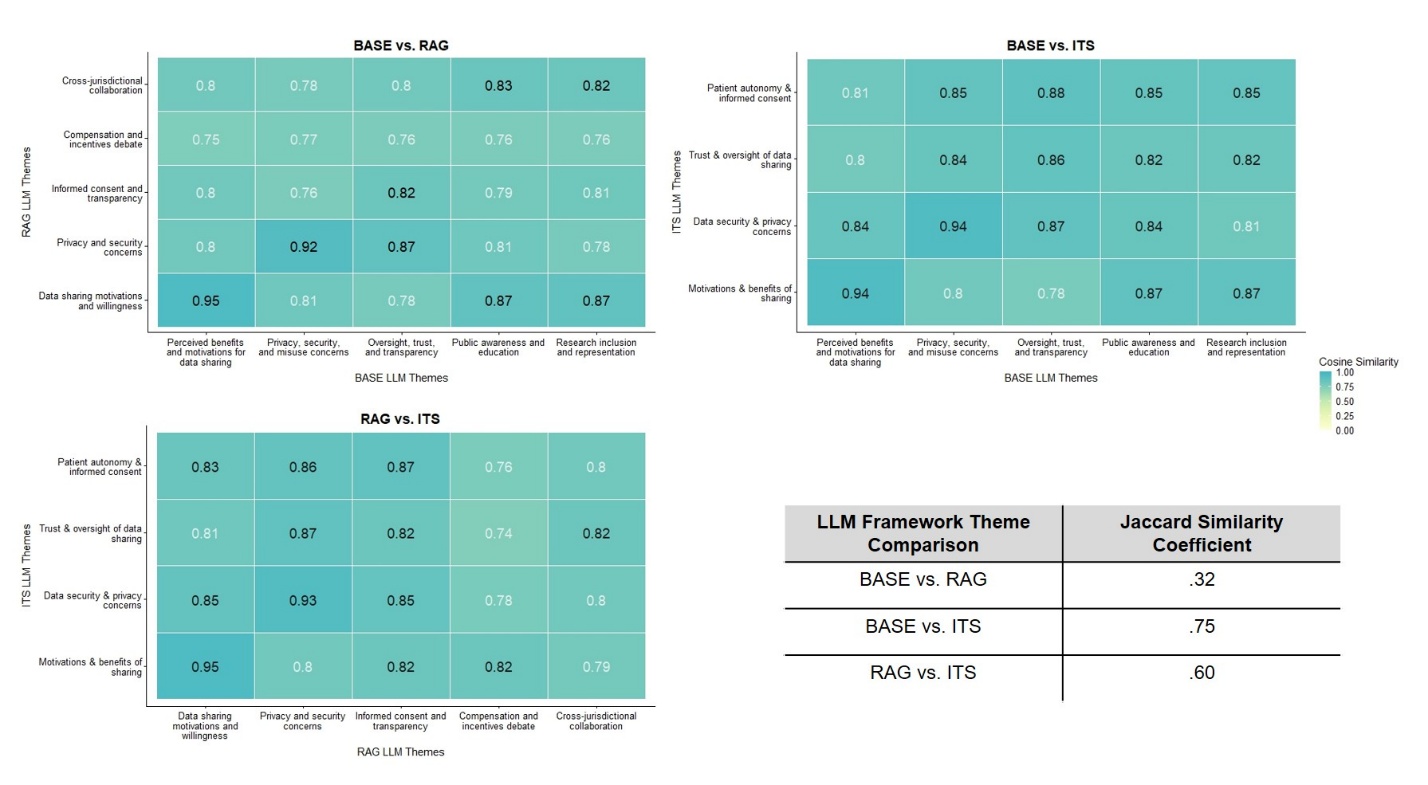
**

**Fig B. BASE prompt framework procedure.** Procedural flowchart outlining the sequential steps for BASE theme generation, and corresponding prompt text used for each step (where applicable).

**
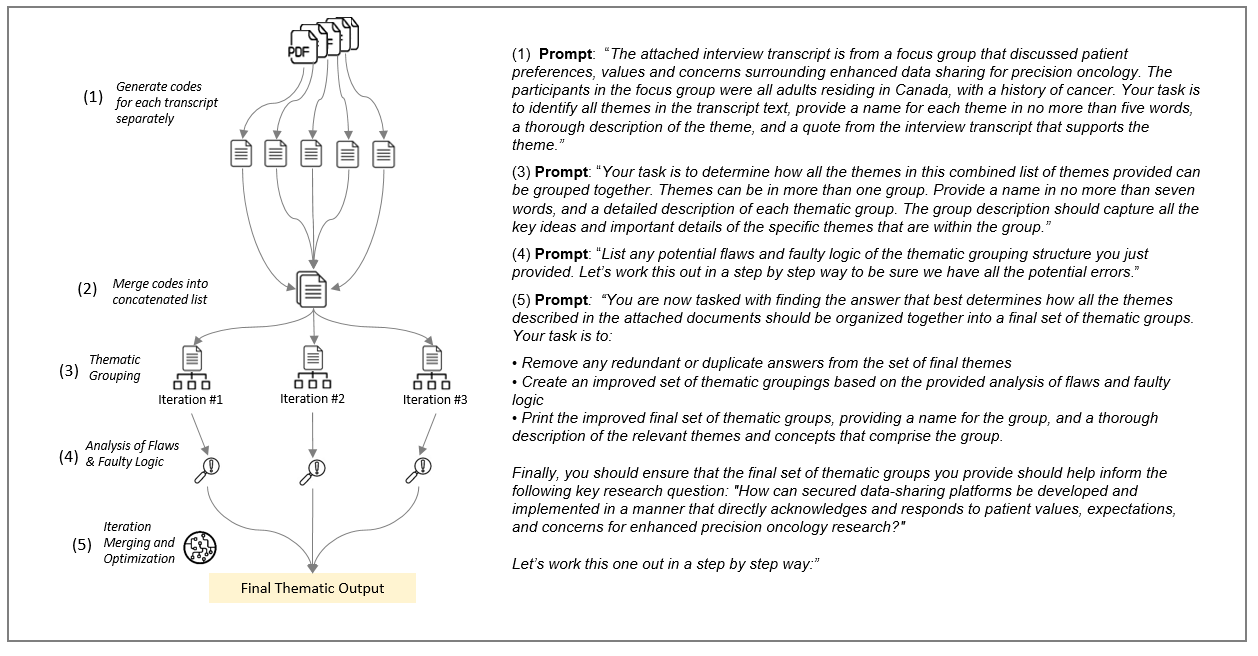
**

**Fig C. RAG prompt framework procedure.** Procedural flowchart outlining the sequential steps for RAG theme generation, and corresponding prompt text used for each step (where applicable).

**
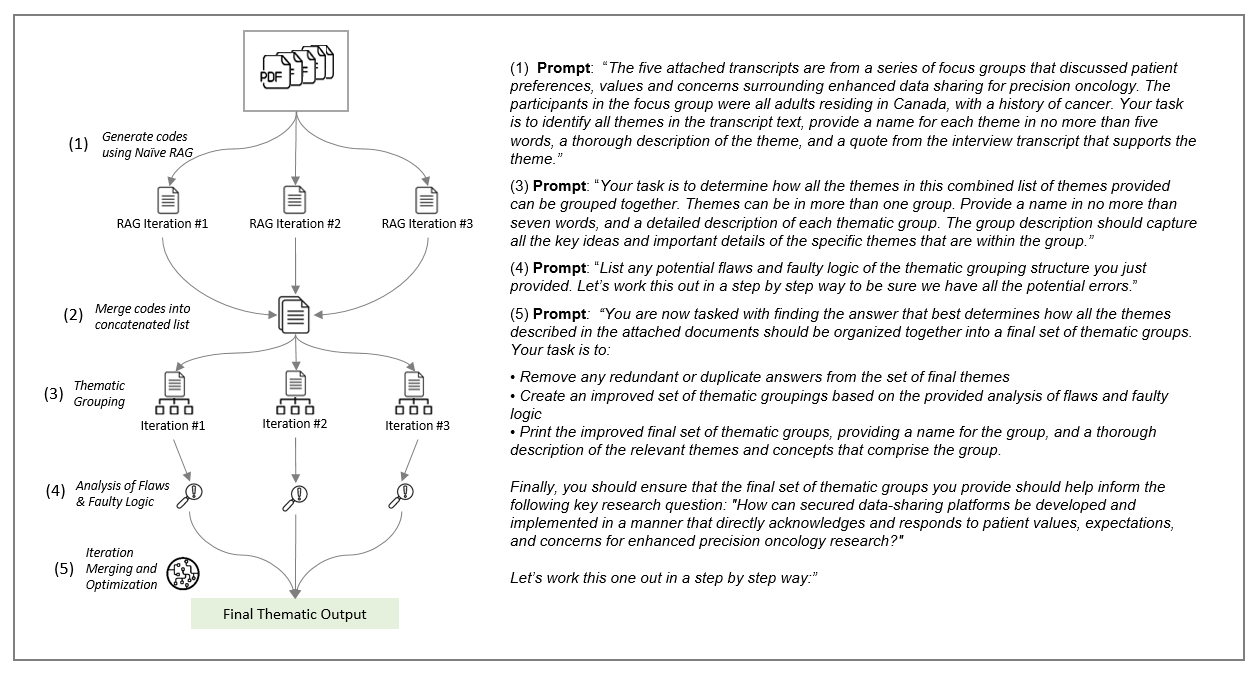
**

**Fig D. ITS prompt framework procedure.** Procedural flowchart outlining the sequential steps for ITS theme generation, and corresponding prompt text used for each step (where applicable).

**
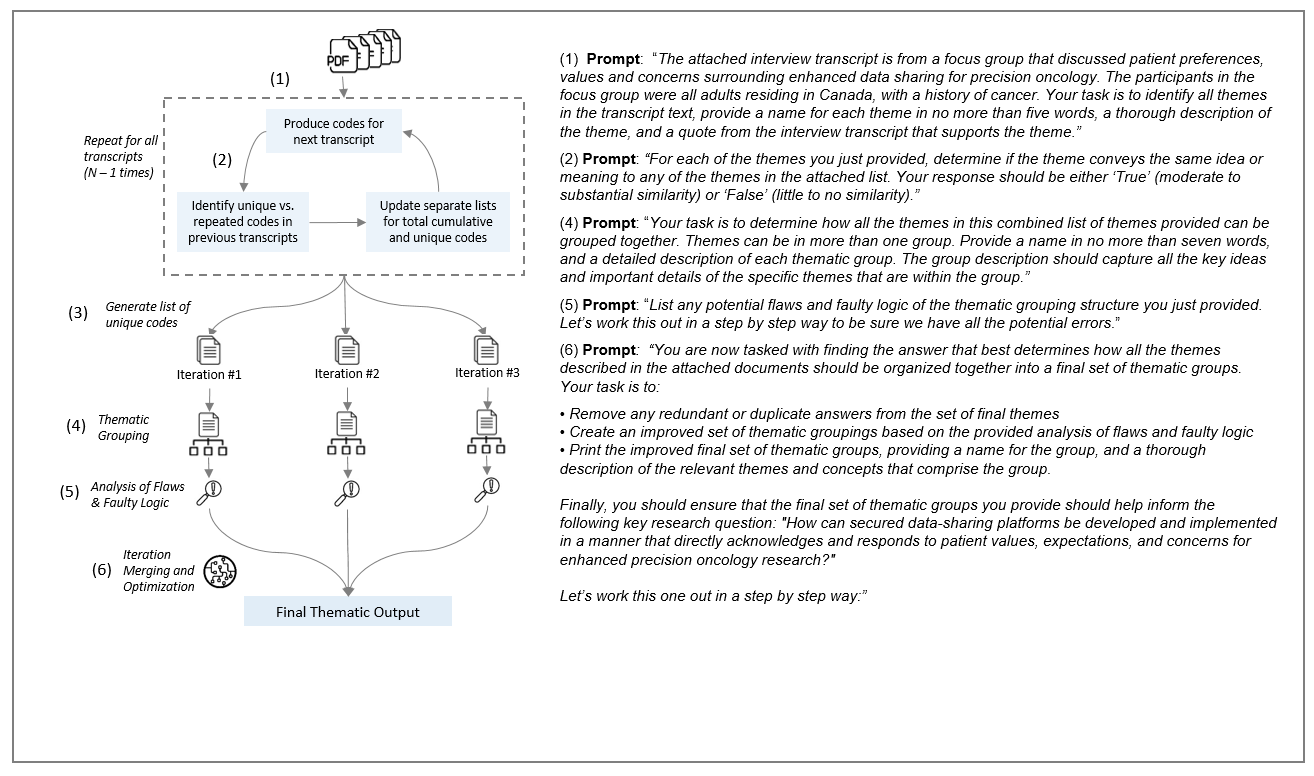
**

**Fig E. Inductive saturation ratio plot for ITS prompt framework.** Inductive saturation ratio plotted for each of the three initial coding three iterations for ITS, where Inductive Saturation Ratio = Unique Codes / Total Codes. The randomized order of transcripts is denoted for each iteration, where T1 is Transcript 1, T2 is Transcript 2, etc.


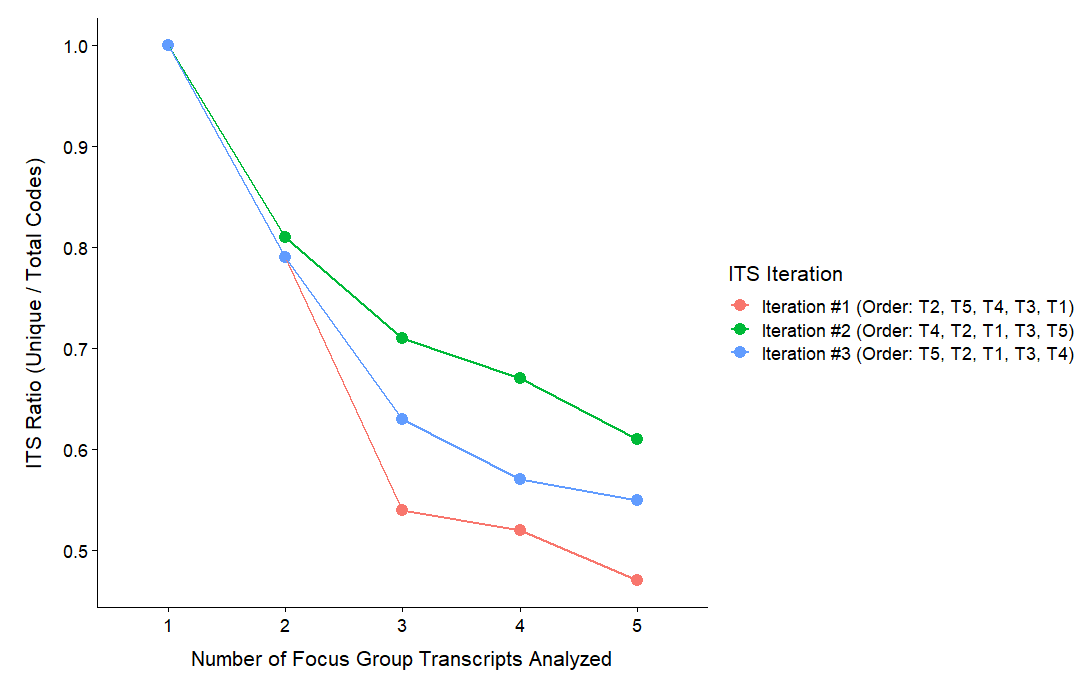


**Fig F. Example intermediate thematic grouping LLM output.** Prompt input and LLM output from our BASE framework (Smart GPT Thematic Analysis) for Thematic Grouping (step iii.), Iteration #1.

**
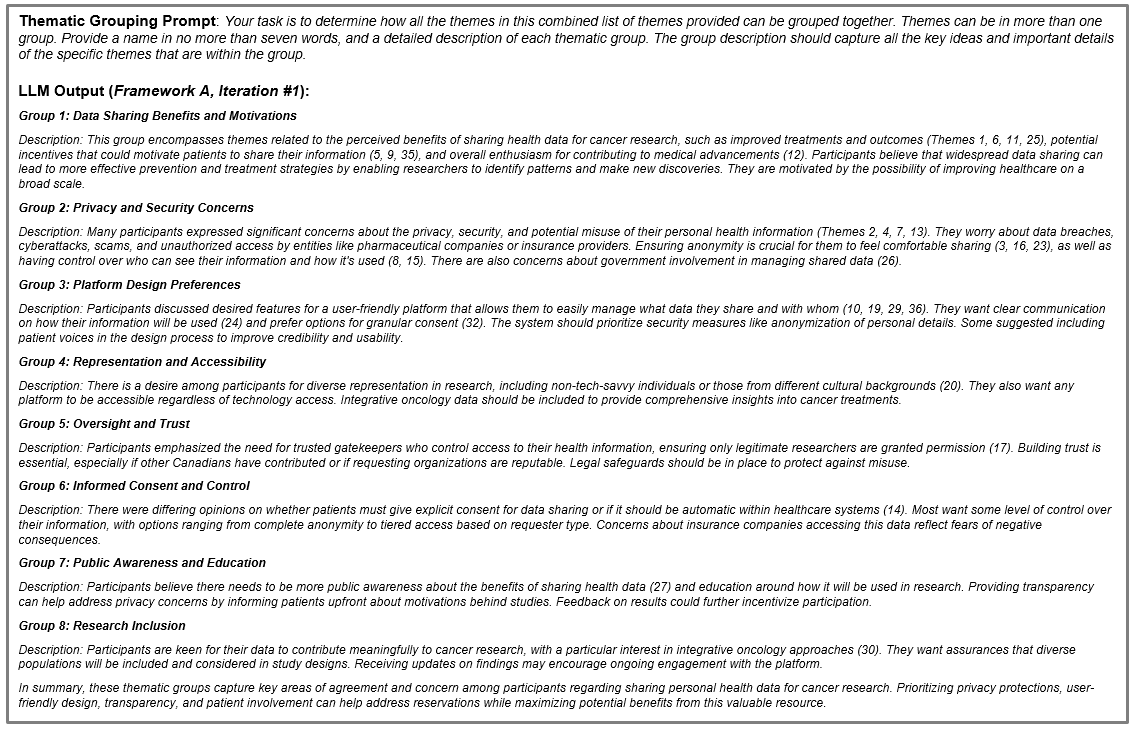
**

**Fig G. Example analysis of flaws and faulty logic LLM output.** Prompt input and LLM output from our BASE framework (Smart GPT Thematic Analysis) for Analysis of Flaws & Faulty Logic (step iv.), Iteration #1.

**
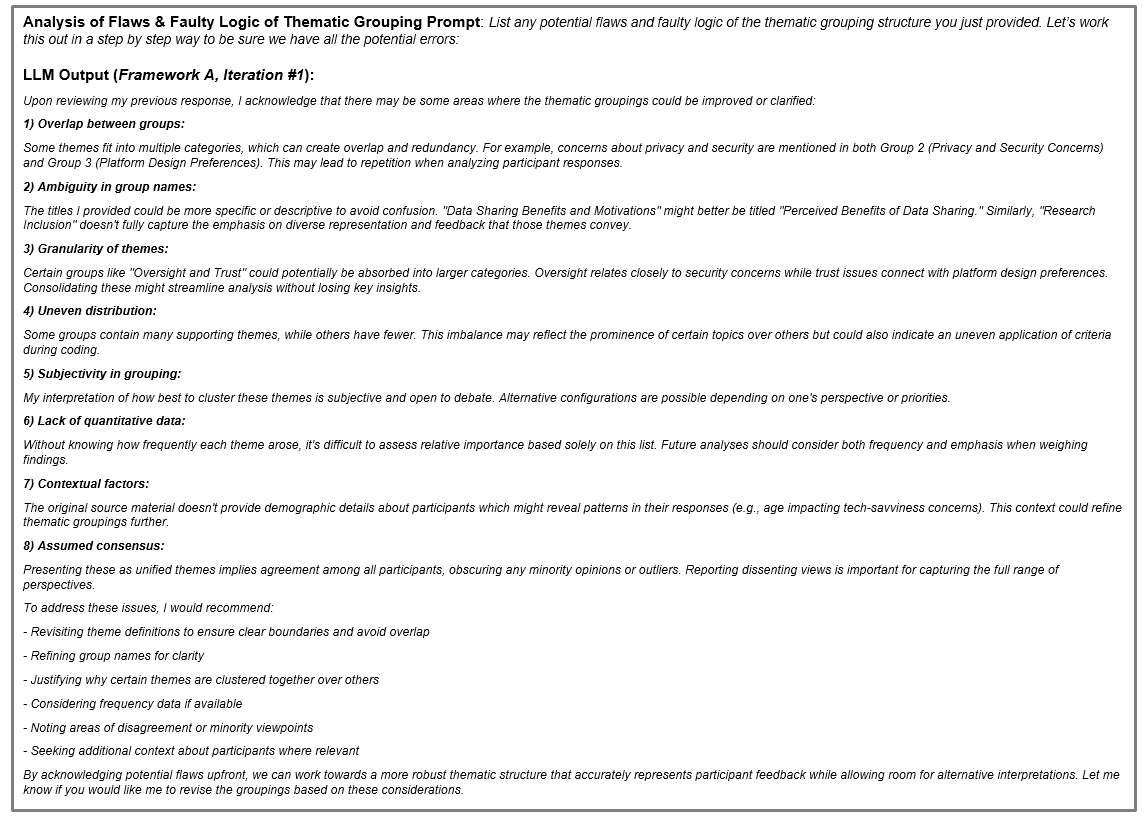
**
